# Adjustment of the multi-biomarker disease activity score to account for age, sex and adiposity in patients with rheumatoid arthritis

**DOI:** 10.1093/rheumatology/key367

**Published:** 2018-12-24

**Authors:** Jeffrey R Curtis, Darl D Flake, Michael E Weinblatt, Nancy A Shadick, Mikkel Østergaard, Merete Lund Hetland, Cecilie Heegaard Brahe, Yong Gil Hwang, Daniel E Furst, Vibeke Strand, Carol J Etzel, Dimitrios A Pappas, Xingbin Wang, Ching Chang Hwang, Eric H Sasso, Alexander Gutin, Elena Hitraya, Jerry S Lanchbury

**Affiliations:** 1 Division of Clinical Immunology and Rheumatology, University of Alabama at Birmingham, Birmingham, AL; 2 Myriad Genetics Inc., Salt Lake City, UT; 3 Department of Rheumatology, Brigham and Women’s Hospital, Boston, MA, USA; 4 Copenhagen Center for Arthritis Research, Center for Rheumatology and Spine Diseases, Centre For Head and Orthopedics, Rigshospitalet, Glostrup, and Department of Internal Medicine, University of Copenhagen, Copenhagen, Denmark; 5 Department of Medicine, University of Pittsburgh, Pittsburgh, PA, USA; 6 David Geffen School of Medicine at UCLA, UCLA, Los Angeles; 7 Division of Immunology/Rheumatology, Stanford University, Palo Alto, CA, USA; 8 Anderson Cancer Center, University of Texas School of Public Health, Houston, TX, USA; 9 Division of Rheumatology, Columbia University, New York, NY, USA; 10 Crescendo Bioscience Inc., South San Francisco, CA, USA

**Keywords:** biomarker, disease activity, leptin, MBDA, multi-biomarker disease activity, radiographic progression, rheumatoid arthritis, Vectra DA

## Abstract

**Objective:**

To develop and evaluate an adjusted score for the multi-biomarker disease activity (MBDA) test to account for the effects of age, sex and adiposity in patients with RA.

**Methods:**

Two models were developed to adjust MBDA score for age, sex and adiposity, using either serum leptin concentration or BMI as proxies for adiposity. Two cohorts were studied. A cohort of 325 781 RA patients who had undergone commercial MBDA testing and had data for age, sex and serum leptin concentration was used for both models. A cohort of 1411 patients from five studies/registries with BMI data was used only for the BMI-adjusted MBDA score. Univariate and multivariate linear regression analyses evaluated the adjusted MBDA scores and conventional clinical measures as predictors of radiographic progression, assessed in terms of modified total Sharp score (ΔmTSS).

**Results:**

Two models were developed, based on findings that MBDA score was higher in females than males and increased with age, leptin concentration and BMI. In pairwise regression analyses, the leptin-adjusted (*P* = 0.00066) and BMI-adjusted (*P* = 0.0027) MBDA scores were significant independent predictors of ΔmTSS after adjusting for DAS28-CRP, whereas DAS28-CRP was not, after adjusting for leptin-adjusted (*P* = 0.74) or BMI-adjusted (*P* = 0.87) MBDA score. Moreover, the leptin-adjusted MBDA score was a significant predictor of ΔmTSS after adjusting for the BMI-adjusted MBDA score (*P* = 0.025) or the original MBDA score (0.027), whereas the opposite was not true.

**Conclusion:**

Leptin-adjusted MBDA score significantly adds information to DAS28-CRP and the original MBDA score in predicting radiographic progression. It may offer improved clinical utility for personalized management of RA.

**Rheumatology key messages**

- An adjusted MBDA score has been developed for use in RA patients.
- The adjusted MBDA score has superior performance *vs* the original MBDA score for RA patients.

## Introduction

Various metrics have been developed to monitor disease activity in patients with RA and support the goal of achieving remission. These include combinations of clinical measures (eg, the Clinical Disease Activity Index (CDAI)) and clinical and laboratory measures (e.g. DAS28-CRP) [1]. The multi-biomarker disease activity (MBDA) blood test measures 12 proteins to produce a validated score, on a scale of 1–100, that represents the level of disease activity in patients with RA [2, 3]. The MBDA score correlates with DAS28-CRP [2] and is a predictor of radiographic progression in patients treated with biologic and non-biologic DMARDs [4–7].

The effects of variability in age, sex and adiposity on the MBDA score are of interest because levels of inflammation generally increase with age, even in the absence of clinical disease, male *vs* female sex might differentially affect the MBDA score, and adipose tissue can secrete or respond to component proteins of the MBDA score, including IL-6, leptin and TNF receptor-I [8, 9]. Thus, adiposity is a potential confounder of the relationship between the MBDA score and RA disease activity or radiographic progression.

In this study we evaluated the effects of variability in age, sex and adiposity on the MBDA score. To account for the effects of adiposity, we used two surrogates to derive two metrics – the leptin-adjusted MBDA score and the BMI-adjusted MBDA score. These adjusted MBDA scores were evaluated for association with radiographic progression and compared with each other and with conventional clinical and clinical/laboratory measures of disease activity.

## Methods

### Study populations

Three cohorts were used in these analyses. Baseline clinical and demographic characteristics and data availability for the healthy normal cohort (*n* = 318), the RA clinical trial/registry cohort (*n* = 1411) and the commercial RA cohort (*n* = 325 781), and for the five cohorts comprising the RA clinical trial/registry cohort (Corrona-CERTAIN, InFoRM, RACER, OPERA and BRASS), are described in Supplementary Tables S1 and S2, available at *Rheumatology* online, and the accompanying supplemental text, available at *Rheumatology* online. Numbers of patients studied here may differ from previously published numbers due to selection for completeness of BMI, MBDA score and radiographic data.

All patients in the healthy normal cohort and the clinical trial/registry cohort signed voluntary informed consent for their respective protocols. All information for patients in the commercial cohort was obtained during the course of standard healthcare operations as part of clinical testing. All patient information was de-identified for analysis and no additional information was obtained from patients or providers. All data analyzed from the clinical trial/registry cohort are from published studies that were previously approved by an institutional review board.

### Clinical and radiographic measures

A measure designated DAS28* was calculated as DAS28, using joint counts and patient global assessment, but without an ESR or CRP component. DAS28* was devised to provide a clinically-based composite measure of disease activity for comparison with the adjusted MBDA scores (see below). DAS28* thus resembles DAS28-CRP, which was used to validate the original MBDA score [2], without sharing any component with the MBDA score. An analogous approach has been used previously [2, 10].

Radiographic data were available only for the OPERA and BRASS cohorts. All patients from OPERA with radiographs at baseline and one year were included here. The BRASS cohort included all patients for whom radiographs had been obtained within 6 months of the baseline clinic visit and 9 months to 3 years after the first radiograph. Because BRASS obtained radiographs only for hands and wrists, for which the maximal modified total Sharp score (mTSS) is 280, mTSS values from BRASS were multiplied by 1.6 (i.e. 448/280) for the present analyses. This assumption was shown to be correct by an orthogonal regression analysis of the OPERA cohort, which found that the factor by which mTSS from hands and wrists in OPERA needed to be multiplied to equal the mTSS for all joints was 1.56 (1.03, 2.09) at baseline and 1.64 (1.17, 2.11) at one year. Change (Δ) in mTSS was calculated as the difference between mTSS at follow-up and baseline, divided by the time in years between the two radiographs.

All clinical, laboratory and radiographic data were treated as observed data, without imputation for missing data.

### MBDA score

De-identified serum specimens were tested in the Clinical Laboratory Improvement Amendment-certified clinical laboratory of Crescendo Bioscience (South San Francisco, CA, USA). A multiplexed, sandwich immunoassay (Mesoscale Discovery, Rockville, MD, USA) was used to measure concentrations of the 12 MBDA protein biomarkers: vascular cell adhesion molecule 1, epidermal growth factor, VEGF alpha, IL-6, TNF receptor-I, MMP-1, MMP-3, YKL-40, leptin, resistin, serum amyloid A and CRP. Concentration values were combined using a previously validated algorithm that generates an integer score on a scale of 1–100 [2]. Samples were tested from 2012 to 2017, using the same immunoassay instruments, reagents and algorithm as those used for the Vectra DA commercial test manufactured by Crescendo Bioscience.

### Leptin-adjusted MBDA score

Using the commercial RA cohort (*n* = 325 781), a linear model was fit with the original MBDA score as the response variable and age, sex, serum leptin concentration and their significant (α = 0.01) interactions as predictors. The relationship between MBDA score and serum leptin was expressed by power-transforming leptin concentration to maximize the likelihood of the linear fittings.

The formula for determining the leptin-adjusted MBDA score from the original MBDA score was established by subtracting the effects of age, sex and leptin concentration on the original MBDA score in the commercial cohort and adding a constant (C) that made the mean of the adjusted MBDA scores in the commercial cohort equal to the mean of the original MBDA scores. Thus: leptin-adjusted MBDA score = [original MBDA score – (effects of age, sex and leptin) + C]. The leptin-adjusted MBDA score is rounded to the nearest integer and set to minimum/maximum values of 1 or 100 if the calculated value is <1 or >100, respectively.

### BMI-adjusted MBDA score

BMI data were not available for patients in the commercial cohort. Thus, we used a two-step process to estimate the effects of age, sex and BMI on the MBDA score. First, using the commercial cohort (*n* = 325 781), a linear model was fit with MBDA score as the response variable, and age, sex and the interaction between age and sex as predictors. The corresponding estimated effects of age, sex and their interaction were then subtracted from MBDA scores of patients in the clinical trial/registry cohort (*n* = 1411), to create intermediary MBDA scores. The next step was to estimate the effect of BMI on the intermediary MBDA score for males and females separately in the clinical trial/registry cohort, for which BMI data were available, by fitting a linear model of the intermediary MBDA score that had BMI as the only predictor.

The final formula for determining the BMI-adjusted MBDA score from the original MBDA score was established by subtracting the effects of age, sex and BMI and adding a constant, for males and females, respectively, that made the mean value of the adjusted MBDA scores in the clinical trial/registry cohort equal to that of the original MBDA scores. Thus: BMI-adjusted MBDA score = [original MBDA score – (effects of age, sex and BMI) + C], where the effects of age, sex and BMI were determined stepwise in two cohorts, as explained above, C depended on sex, and the score is rounded to the nearest integer and set to minimum/maximum values of 1 or 100 if the calculated value is <1 or >100, respectively.

### Evaluation of the adjusted MBDA scores

The original MBDA score, the leptin-adjusted MBDA score and the BMI-adjusted MBDA score were compared in terms of their respective associations with disease activity in the clinical trial/registry cohort. Univariate regression models were fit with DAS28* (DAS28 without CRP or ESR) as the response variable and each MBDA score as predictors. Multiple linear regression models of DAS28* were fit with pairs of MBDA scores as concurrent predictors.

The association of radiographic progression with each MBDA score (original, leptin-adjusted, BMI-adjusted) and with other variables was assessed in the combined OPERA and BRASS cohorts using univariate linear regression models of ΔmTSS. To compare the three types of MBDA score in terms of their respective associations with radiographic progression, we performed pairwise linear regression analyses that included each pair of MBDA scores as covariates in a model predicting ΔmTSS. In both the univariate and bivariate models, F-tests were used to calculate *P*-values for each predictor.

To further elucidate the performance of the disease activity measures, descriptive analyses were performed without statistical testing to determine absolute risk for radiographic progression (ΔmTSS >5 units per year) for the low, moderate or high categories of leptin-adjusted MBDA score, original MBDA score, CRP, DAS28-CRP, Simplified Disease Activity Index (SDAI) and CDAI. For each of these measures, relative risks were determined as: (% patients with radiographic progression in the moderate or high disease activity category) divided by (% patients with radiographic progression in the low category). Established categories were used for MBDA score [2], DAS28-CRP [11], SDAI and CDAI [1]. CRP categories were chosen on the basis of 3 mg/l being a threshold for cardiovascular risk [12], 10 mg/dl being in the ACR/EULAR Boolean definition of remission in RA [13], and for consistency with prior publications of MBDA data [4–6, 14].

All analyses were performed with R v3.3.1 or a newer version [15]. No corrections were made for multiple comparisons.

## Results

### Leptin as a proxy for body mass index in rheumatoid arthritis

**Fig. 1:**
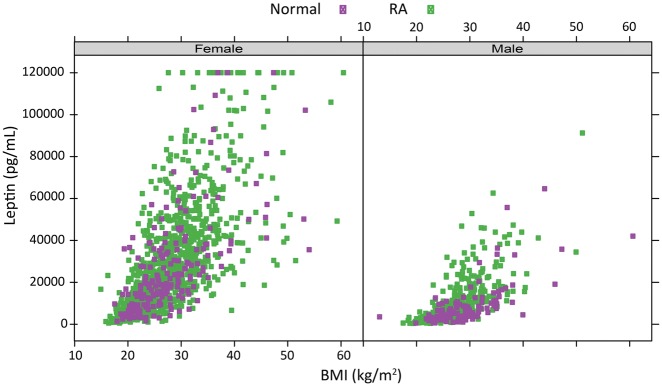
Relationship between BMI and serum leptin concentration for patients with RA or subjects without RA Patients with RA: *n* = 1411 total; 1098 female, 313 male; Subjects without RA (Normal): *n* = 318 total; 203 female, 115 male. RA patients are from the clinical trial/registry cohort. Subjects without RA are from the healthy normal cohort. The upper limit of quantitation for leptin concentration in the MBDA test is 120 ng/ml, as observed here for 26 patients (1.5%): 23 with RA, 3 without RA. MBDA: multi-biomarker disease activity.

To evaluate leptin as a proxy for adiposity, we compared serum leptin concentration and BMI in a cohort of healthy subjects without RA and a cohort of patients with RA from five clinical trials/registries. Leptin showed a significant positive correlation with BMI in healthy females (r = 0.66, *P* = 7.1 × 10^−27^) and males (r = 0.69, *P* = 1.4 × 10^−17^) and in patients with RA (r = 0.69, *P* = 2.7 × 10^−155^, females; r = 0.69, *P* = 4.7 × 10^−46^, males) (Fig. 1). We therefore used serum leptin concentration as a proxy for adiposity in RA.

### Leptin-adjusted MBDA score

**Fig. 2:**
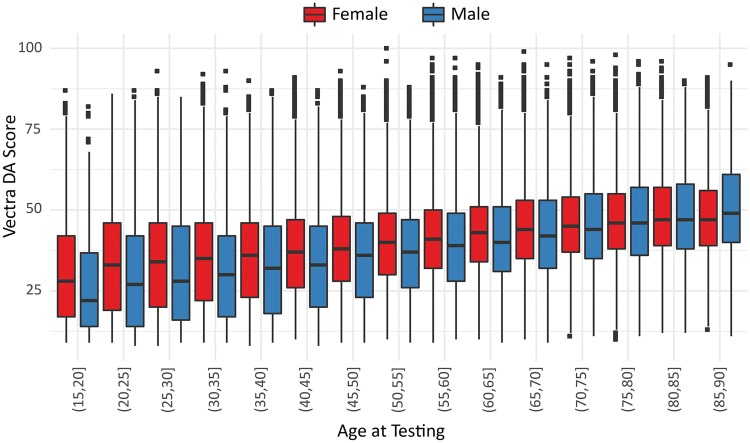
Relationship between age and MBDA score in 325 781 RA patients, shown separately for females (*n* = 256 470) and males (*n* = 69 311) Horizontal lines are medians; boxes define the 25th–75th percentiles (IQR), vertical lines define 1.5×IQR, square dots represent individual patients beyond 1.5×IQR. MBDA: multi-biomarker disease activity; IQR: interquartile range.

In the cohort of 325 781 RA patients who had received commercial MBDA testing, MBDA scores increased with age in a continuous, linear fashion − more steeply in males than females (Fig. 2). MBDA scores tended to be greater among younger females than younger males and were similar between sexes among older patients (e.g. age >75 years) (Fig. 2).

MBDA scores tended to be greater among patients with greater serum leptin concentrations, especially among younger patients (e.g. age 15−30 years) (Supplementary Fig. S1, available at *Rheumatology* online). This association was less pronounced in older age groups and was nearly absent in the oldest group (age 75−90 years). The over all relationship between MBDA score and leptin was non-linear and was best expressed exponentially; the exponent of leptin that maximized its ability to predict the MBDA score was 0.58. The pairwise interactions between age, sex and serum leptin concentration were all statistically significant predictors of MBDA score (Table 1).

**Table 1:** The effects of age, sex and leptin on the MBDA score

Variable	Coefficient (95% CI)	*P*-value^a^
Age	0.437 (0.429, 0.444)	<1.0×10^−15^
Sex (female as reference)	3.31 (2.74, 3.88)	<1.0×10^−15^
Leptin concentration to the 0.58th power	0.0502 (0.0492, 0.0513)	<1.0×10^−15^
Age×Sex	−0.0247 (−0.0338, −0.0155)	1.3×10^−7^
Sex×Leptin	0.00254 (0.00174, 0.00334)	4.1×10^−10^
Leptin×Age	−0.000483 (−0.000500, −0.000465)	<1.0×10^−15^

Effects are shown in terms of magnitude, direction and statistical significance in a cohort of 325 781 RA patients.

a P-values determined with the F-test.

MBDA: multi-biomarker disease activity.

The leptin-adjusted MBDA score was constructed by subtracting the effects of age, sex, leptin concentration and their pairwise interactions from the MBDA score. By combining the adjustments for these interactions with a constant value (33.9) that kept the mean values of the original and leptin-adjusted MBDA scores equal in the commercial cohort, the following formula was derived:

$$\text{Leptin}-\text{adjusted}\text{MBDA}\text{score}=originalMBDAscore-(0.437\times age+3.31\times sex+0.0502\times {leptin}^{0.58}-0.0247\times age\times sex-0.000483\times age\times {leptin}^{0.58}+0.00254\times sex\times {leptin}^{0.58})+33.9$$

where sex is equal to 1 if the patient is male and 0 if female. The leptin-adjusted MBDA score is rounded to the nearest integer, with minimum and maximum values of 1 and 100, respectively.

### BMI-adjusted MBDA score

Because BMI is commonly used as a surrogate for adiposity, we wished to also construct a model for an adjusted MBDA score that used BMI rather than leptin. BMI data were not available for the commercial cohort of 325 781 patients. Therefore, we derived a BMI-adjusted MBDA score in two steps, using two datasets for maximal robustness. First, an intermediary MBDA score was generated by adjusting for age, sex and their interaction with a linear regression equation that was derived from the commercial cohort dataset (*n* = 325 781) and was analogous to the equation generated for the leptin-adjusted MBDA score, without any leptin-related terms.

Next, the clinical trial/registry cohort (*n* = 1411), which included BMI data, was used to adjust the intermediary MBDA score for BMI and its interactions with age and sex. The effect of BMI in the clinical trial/registry cohort was significantly different between sexes: −0.203 (−0.575, 0.170) per BMI unit in males and 0.384 (0.233, 0.535) per BMI unit in females (interaction *P* = 0.0043).

A final formula for the BMI-adjusted MBDA score was created by combining the adjustments for age and sex, derived from the commercial patient cohort, with the adjustment for BMI, derived from the clinical trial/registry cohort, and adding a constant value (25.7) that kept the mean BMI-adjusted MBDA score in the clinical trial/registry cohort, for each sex, the same as for the original MBDA score:

BMI-adjustedMBDAscore=originalMBDAscore-(0.267×age+10.9×sex+0.384×BMI+0.0667×age×sex-0.587×sex×BMI)+25.7

where sex is equal to 1 if the patient is male and 0 if female. The BMI-adjusted MBDA score is rounded to the nearest integer, with minimum and maximum values of 1 and 100, respectively.

### Evaluation of the leptin-adjusted and BMI-adjusted MBDA scores

#### Association with DAS28*

To examine which biomarker-based measures correlated most strongly with clinical assessment of RA disease activity, the associations with DAS28* for CRP and each MBDA score were assessed in the same clinical trial/registry cohort (*n* = 1411) that was used for BMI adjustment. Each measure was significantly correlated with DAS28*, with r = 0.34 (*P* = 1.3 × 10^−39^) for CRP (using the base-10 logarithm of CRP), r = 0.38 (*P* = 2.7 × 10^−48^) for the original MBDA score, r = 0.39 (*P* = 2.3 × 10^−52^) for the BMI-adjusted MBDA score, and r = 0.40 (*P* = 4.6 × 10^−54^) for the leptin-adjusted MBDA score.

In pairwise linear regression models with DAS28* as the response variable, the leptin-adjusted MBDA score was significantly associated with DAS28* (*P* = 1.6 × 10^−16^ when adjusted for base-10 logarithm of CRP; *P* = 2.3 × 10^−7^ when adjusted for the original MBDA score), whereas CRP and the original MBDA score were not, when each was adjusted for the leptin-adjusted MBDA score, (*P* = 0.18, *P* = 0.60, respectively). A similar finding was obtained when the BMI-adjusted MBDA score (*P* = 9.2 × 10^−15^ and 1.5 × 10^−5^, respectively) was included in the model with CRP (*P* = 0.21) or the original MBDA score (*P* = 0.93). When the adjusted MBDA scores were combined in the same model, the leptin-adjusted score (*P* = 0.0048) was a significant predictor of DAS28*, whereas the BMI-adjusted score (*P* = 0.71) was not.

#### Association with radiographic progression

Radiographic data were available from the combined OPERA and BRASS cohorts. In univariate analyses of these cohorts, the measure with the numerically strongest association with ΔmTSS per year, i.e. with radiographic progression, was the leptin-adjusted MBDA score (Table 2). This association, as the F-statistic, was slightly larger than was observed for seropositivity, the BMI-adjusted MBDA score or the original MBDA score and was much larger than for clinically-based measures (DAS28-CRP, SDAI, CDAI) (Table 2). The three MBDA scores had similar coefficients (0.024, 0.022, 0.021).

**Table 2:** Association of disease activity measures with radiographic progression

	Variable	N^a^	Coefficient^b^	F-statistic	*P*-value
Leptin-adjusted MBDA score		555	0.024 (0.012, 0.035)	17.0	0.000042
Seropositive (RF &/or anti-CCP)		420/555	0.93 (0.46, 1.41)	14.8	0.00013
BMI-adjusted MBDA score		555	0.022 (0.011, 0.033)	14.4	0.00016
Original MBDA score		555	0.021 (0.009, 0.032)	12.9	0.00036
BMI		555	−0.071 (−0.11, −0.03)	10.9	0.0010
log_10_(CRP)		555	0.41 (0.10, 0.72)	6.8	0.0093
Baseline mTSS		555	0.0033 (0.0005, 0.0062)	5.3	0.022
log_2_(Disease duration + 1)		401	0.16 (0.016, 0.30)	4.8	0.030
DAS28-CRP		536	0.14 (0.012, 0.28)	4.6	0.032
SDAI		533	0.013 (0.001, 0.025)	4.3	0.040
CDAI		533	0.013 (0.000, 0.026)	3.9	0.049
DAS28*		536	0.14 (−0.02, 0.29)	3.1	0.079
Male		123/555	−0.31 (−0.80, 0.19)	1.5	0.23
Smoking status	Never	240/478	Reference	0.79	0.46
	Former	160/478	0.22 (−0.27, 1.03)		
	Current	78/478	0.38 (−0.29, 0.73)		
Age		555	0.0025 (−0.013, 0.018)	0.09	0.76

Univariate linear regression was used to evaluate the association of baseline demographic and disease-related variables with ΔmTSS (degree of radiographic progression) in the combined OPERA and BRASS cohorts. Results are in descending order of statistical significance.

a Patients within the total group that had suitable radiographic data (*n* = 555) for whom baseline data were available for the indicated variable. Ratios indicate the number of patients in the indicated category and the total number with data available for that variable.

b Coefficients for continuous variables (i.e. all except seropositivity, male and smoking status) represent slope of the linear regression line, expressed as units of ΔmTSS per one-unit change in the indicated variable. anti-CCP: anti-cyclic citrullinated peptide; DAS28*: DAS28 with no CRP or ESR component; MBDA: multi-biomarker disease activity; ΔmTSS: change in modified total Sharp score.

Because the measures in Table 2 are related, we compared them by using pairwise linear regression models to test the ability of a measure to predict ΔmTSS independently of other measures. In these analyses, the leptin-adjusted (*P* = 0.00066) and BMI-adjusted (*P* = 0.0027) MBDA scores were statistically significant predictors of radiographic progression after adjusting for DAS28-CRP. However, DAS28-CRP was not a significant predictor after adjusting for either of these MBDA scores (*P* = 0.74 and *P* = 0.87, respectively). The leptin-adjusted MBDA score predicted progression after adjusting for the original MBDA score (*P* = 0.027) or the BMI-adjusted MBDA score (0.025), whereas neither the original MBDA score nor the BMI-adjusted MBDA score predicted progression after adjusting for the leptin-adjusted MBDA score (*P* = 0.34 and *P* = 0.11, respectively). Thus, the leptin-adjusted MBDA score had a statistically significantly stronger and independent correlation with radiographic progression, compared with the original MBDA score or the BMI-adjusted MBDA score.

#### Absolute and relative risk for radiographic progression

In descriptive analyses, the rates of radiographic progression (ΔmTSS >5) tended to be numerically lowest in low disease activity groups for MBDA score and other measures of disease activity, and highest in the high disease activity groups, except for SDAI, for which the low and moderate categories had similar rates of radiographic progression, as was also observed for CDAI (Table 3). The lowest rate of radiographic progression in a low disease activity group, and the greatest difference between rates in the low *vs* high groups, were observed with the leptin-adjusted MBDA score, for which 1.1%, 3.9% and 9.3% of patients in the low, moderate and high groups, respectively, had progression. Relative risk for radiographic progression was numerically greatest in the high category of leptin-adjusted MBDA score, followed by the high category of original MBDA score, and was non-significant for DAS28-CRP, SDAI and CDAI (Table 3).

**Table 3:** Proportion of patients with radiographic progression (RP) and relative risk for patients according to disease activity

Disease activity measure	Disease activity Category	Thresholds	*n*	Proportion of patients with RP (95% CI^a^)	RR^b^^,c^ (Moderate *vs* Low)	RR^b^^,^^c^ (High *vs* Low)
Leptin-adjusted MBDA score	Low	<30	90	1.1% (0%, 6.0%)	3.53 (0.43, 28.85)	8.38 (1.15, 60.8)
	Moderate	30–44	153	3.9% (1.5%, 8.3%)		
	High	>44	290	9.3% (6.2%, 13.3%)		
MBDA score	Low	<30	111	1.8% (0.2%, 6.4%)	1.93 (0.38, 9.75)	5.39 (1.3, 22.29)
	Moderate	30–44	144	3.5% (1.1%, 7.9%)		
	High	>44	278	9.7% (6.5%, 13.8%)		
CRP	Low	<3 mg/l	195	2.6% (0.8%, 5.9%)	2.54 (0.9, 7.16)	4.15 (1.58, 10.95)
	Moderate	3–10 mg/l	169	6.5% (3.3%, 11.3%)		
	High	>10 mg/l	169	10.7% (6.4%, 16.3%)		
DAS28-CRP	Low	≤2.67	104	5.8% (2.1%, 12.1%)	0.49 (0.12, 1.89)	1.35 (0.57, 3.19)
	Moderate	2.68–4.08	107	2.8% (0.6%, 8.0%)		
	High	>4.09	322	7.8% (5.1%, 11.2%)		
SDAI	Low	≤11	123	4.1% (1.3%, 9.2%)	1.04 (0.33, 3.32)	2.11 (0.82, 5.42)
	Moderate	12–26	142	4.2% (1.6%, 9.0%)		
	High	>26	268	8.6% (5.5%, 12.6%)		
CDAI	Low	≤10	121	4.13% (1.4%, 9.4%)	1.03 (0.30, 3.45)	1.98 (0.77, 5.06)
	Moderate	11–22	118	4.2% (1.4%, 9.6%)		
	High	>22	294	8.2% (5.3%, 11.9%)		

Proportions are shown for patients in the combined OPERA and BRASS cohorts by disease activity category (low, moderate or high), based on the indicated disease activity measures.

a Exact binomial CIs were used for rates of RP.

b Wald-type CIs were used for RR.

c RR defined as a ratio of rates of RP in the corresponding disease activity categories. BRASS: Brigham and Women’s Rheumatoid Arthritis Sequential Study; OPERA: Observational Pharmaco-Epidemiology Research & Analysis; RP: radiographic progression (defined as ΔmTSS >5 units per year); RR: relative risk; MBDA: multi-biomarker disease activity.

### Clinical relevance of leptin-adjusted MBDA score

**Fig. 3:**
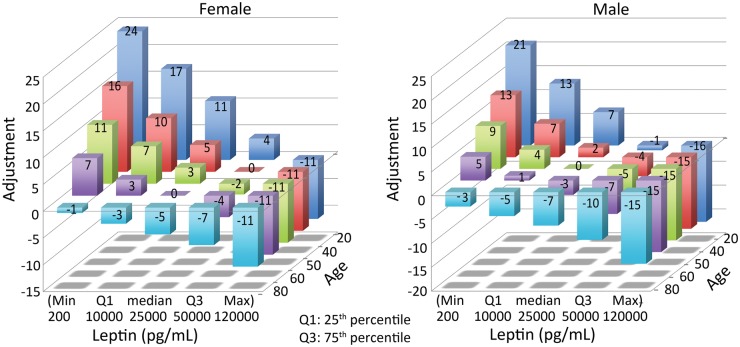
Difference between the adjusted and original MBDA score based on age and leptin Magnitude of adjustments made by the leptin-adjusted MBDA formula to the original MBDA score, based on patient age and leptin concentration, are shown separately for females and males. For leptin, Q1=25th percentile; Q3=75th percentile, based on the distribution of leptin concentrations in the commercial RA cohort. MBDA: multi-biomarker disease activity.

To examine the extent to which leptin-adjustment alters the original MBDA score, three analyses were performed. Figure 3 shows that, for the many combinations of age and serum leptin that are possible across a broad range of each variable, the resulting differences between the leptin-adjusted MBDA score and the original MBDA score vary considerably, with females and males having different distributions. For both sexes, however, young age or low leptin concentration lead to the greatest increases of the MBDA score while old age or high leptin concentration lead to the greatest decreases. It should be noted that these score changes are direct effects of age, leptin and sex in the formula for the leptin-adjusted MBDA score, and are not affected by other characteristics of a given patient.

As a real-life example of the consequences of using the leptin-adjusted MBDA score, we found that, in the commercial RA cohort (*n* = 325 781), it differed from the original MBDA score by -9 to +11 points for 90% of patients (Supplementary Fig. S2, available at *Rheumatology* online). Of patients with an original MBDA score in the low category, the leptin-adjusted MBDA score reclassified 24% to the moderate category (Supplementary Table S3, available at *Rheumatology* online); of those in the moderate category, 9% and 12% were reclassified to the low and high categories, respectively; of those in the high category, 21% were reclassified to the moderate category.

## Discussion

The reliability of measurements of disease activity in RA patients who have high or low BMI [16] or are elderly [17] has been controversial, and differential effects among females and males have been suggested from observations in clinical practice [18]. The MBDA score has been validated as an objective measure of RA disease activity and shown to be a stronger predictor of RP than conventional measures. Systemic inflammation tends to increase with age [19], and adipose tissue produces adipokines and other factors with inflammatory potential. Therefore, we studied the relationship between the MBDA score and age, sex and adiposity. We used serum leptin concentration and BMI, respectively, as surrogates for adiposity in the absence of data from direct measurement of total body fat mass. With this approach, we derived two formulas that improved the performance of the MBDA score by adjusting it for age, sex and either leptin or BMI, with the best performance observed in the leptin-adjusted MBDA score.

A strength of the study was the availability of a cohort of 325 781 RA patients who had been tested commercially for the MBDA score as part of routine clinical practice. This cohort provided considerable statistical power in detecting and accounting for effects of age, sex and adiposity. When tested in a separate, clinical trial/registry cohort of 1411 RA patients, the leptin-adjusted MBDA score, BMI-adjusted MBDA score and original MBDA score were all highly associated with DAS28*, a DAS28 variant based only on joint counts and patient global assessment. Furthermore, the three MBDA tests were directly compared in bivariate modelling, which showed that no significant ability to predict DAS28* remained for the original or BMI-adjusted MBDA scores after adjusting for leptin-adjusted MBDA score. This result means that, of the three versions of MBDA score, the leptin-adjusted MBDA score had the strongest association with clinically-based assessment of RA disease activity.

To further understand the performance of the adjusted MBDA scores and conventional disease activity and laboratory measures, we evaluated their abilities to predict radiographic progression. Leptin-adjusted MBDA score was the strongest individual predictor of new radiographic damage over one year, independently contributing significant information to the original and BMI-adjusted MBDA scores. Seropositivity was the second strongest predictor. In a previous study with a different cohort, the original MBDA score was shown to predict radiographic progression independently of seropositivity, and more strongly [6]. Thus, our findings suggest the leptin-adjusted MBDA score and seropositivity, in combination, may potentially offer a stronger predictor of structural damage than either one alone.

Our adjusting the MBDA score for adiposity might, theoretically, have reduced the ability of the MBDA score to measure disease activity and predict radiographic progression because leptin is a component of the MBDA score and BMI has been found to have an inverse relationship with progression [20]. Leptin contributes to the adjusted MBDA score in two distinct ways. First, leptin and the other MBDA biomarkers act within the algorithm to produce the original MBDA score, as previously. This multi-biomarker effect is specific to the individual patient at the time of testing. The second contribution of leptin is as one of the three variables (age, sex, leptin) used to generate the adjusted MBDA score. In this role, leptin serves as a surrogate for adiposity, to adjust the original MBDA score according to the population-based relationship we established between leptin and the original MBDA score, with consideration for the interactions between leptin, age and sex. This adjustment of the original MBDA score does not alter the contribution leptin made within the MBDA algorithm. Empirically, most effects removed by adjusting the MBDA score for age, sex and either leptin or BMI were probably not due to disease activity because adjustment improved the performance of the MBDA score for predicting clinical disease activity, as DAS28*, and radiographic progression, as ΔmTSS.

Another theoretical concern is that using the clinical trial/registry cohort to evaluate the effect of BMI on the MBDA score and to test the relationship of the BMI-adjusted MBDA score with DAS28* and radiographic progression may have led to overfitting. However, the leptin-adjusted MBDA score, which was developed only with data from the large commercial RA cohort (*n* = 325 781), demonstrated superior performance to the BMI-adjusted MBDA score when each one was evaluated in the clinical trial/registry cohort. It is possible that some patients in the commercial cohort did not have RA. The MBDA score was developed and validated only in RA patients. It is not intended for diagnosis of RA and it is not reimbursed for any condition other than RA. Therefore, the number of patients who might not have had RA should be too small to have meaningfully affected data derived from the commercial cohort.

The low, moderate and high categories for the original MBDA score were established several years prior to this study by translating the DAS28-CRP thresholds to the corresponding MBDA scores based on the linear relationship between the scales of DAS28-CRP and the MBDA score [2]. By this methodology, the disease activity categories remain the same for the adjusted MBDA scores developed here. A direct effect of the formula for leptin-adjusted MBDA score is that the largest changes from the original MBDA score occur when age and leptin concentration are both very high or very low. Most patients, however, are not at these extremes. Accordingly, we found that categorizations were altered for a minority of patients. Among commercial cohort patients whose original MBDA score was in the moderate category, the leptin-adjusted MBDA score kept 79% in the moderate category and reclassified 21% to the high or low categories.

In summary, we developed a leptin-adjusted MBDA score that has significantly improved ability to predict clinical disease activity and radiographic progression, as assessed by DAS28* and ΔmTSS, respectively. These results suggest that the leptin-adjusted MBDA score represents an improvement over both the original MBDA score and commonly used disease activity measures for predicting radiographic progression in RA.

## Supplementary Material

Supplementary Data
