# Supplementary material for: Adjustment of the multi-biomarker disease activity score to account for age, sex and adiposity in patients with rheumatoid arthritis
Source: Rheumatology (Oxford). 2018 Dec 24;58(5):874–83. doi: 10.1093/rheumatology/key367 (PMC6477524; doi:10.1093/rheumatology/key367)
Supplement: Supplementary Data [file key367_supplementary_data.docx]

**SUPPLEMENTAL MATERIAL**

## Healthy Normal Cohort (N=318)

Serum samples from healthy controls were obtained from a commercial source, BioreclamationIVT, (Westbury, NY) and from family members of patients with a cancer diagnosis admitted to the Fox Chase Cancer Center (Philadelphia, PA). Mean age was 53 years (range, 20−80 years); 63% were female. Control subjects had no history of acute or chronic disease and were on no medications other than vitamin supplements or intermittently used non-steroidal anti-inflammatory drugs. All serum samples had been processed within 4 hours of phlebotomy and stored at –70°C prior to MBDA testing.

## 5 Cohorts of Patients with RA from Clinical Trials or Registries (N=1411)

**InFoRM** (**In**dex **Fo**r **R**heumatoid Arthritis **M**easurement). 382 patients from a prospective North American multicenter, longitudinal, observational study of patients with RA were included in this study. Clinical characteristics and laboratory details have been described previously [1].

**RACER** (**R**heumatoid **A**rthritis **C**omparative **E**ffectiveness **R**esearch). Since its inception in February 2010, the RACER registry of the University of Pittsburgh Medical Center (UPMC) has enrolled patients >18 years of age who have been diagnosed with RA by a rheumatologist and were being followed by rheumatologists at UPMC. From a cohort of 740 patients in RACER with DAS28-CRP, CDAI, SDAI, RAPID3 and MBDA data, the present study included the 332 patients for whom BMI data were available, using data from the earliest visit in RACER for which serum samples were available.

**Corrona-CERTAIN** (**C**omparative **E**ffectiveness **R**egistry to Study **T**herapies for **A**rthritis and **I**nflammatory Co**N**ditions) Cohort. The Corrona-CERTAIN study enrolled 2814 patients fulfilling ACR criteria for RA with moderate disease activity (CDAI>10) for whom a change in therapy was planned. Patients were recruited through the CORRONA registry by a network of physicians in private and university hospital clinics in the US [2].  Recruitment to the CERTAIN study was conducted during a routine patient visit, based on the patient having been selected to initiate treatment with a biologic DMARD. The present study included 105 patients who comprised a random subset of all patients who were about to initiate treatment with adalimumab as a first biologic in the Corrona-CERTAIN study. Serum samples were from the Corrona-CERTAIN baseline visit.

**OPERA** (**OP**timized Treatment Algorithm in **E**arly **R**heumatoid **A**rthritis) Cohort. 180 treatment-naïve patients with early RA were recruited through the Departments of Rheumatology in Copenhagen, Herlev, Gråsten, Aarhus, Odense, Silkeborg, Vejle, Hjørring, Aalborg and Viborg, Denmark. Patients were randomized to step-up treatment with oral MTX and either adalimumab (n=89) or placebo (n=91) [3]. Glucocorticoid injections could be administered in up to 4 swollen joints per visit. Radiographs of hands and feet at 0 and 12 months were assessed by Sharp van der Heijde modified total Sharp Score (mTSS). Serum samples for the present study were from OPERA baseline, i.e. prior to initiation of treatment. The 80 (adalimumab) and 88 (placebo) patients with available BMI data and serum samples were included in this study.

**BRASS** (**B**righam **R**heumatoid **A**rthritis **S**equential **S**tudy). 424 RA patients with available data as of February 2017 were studied from an ongoing prospective, observational cohort at the Brigham and Women’s Arthritis Center in Boston, MA. Clinical characteristics and laboratory details have been described previously [4]. Clinical data and serum samples were collected at baseline. Radiographs included hands and wrists only, per BRASS protocol.

**Commercial Clinical Testing Cohort (N=325,781)**

This cohort included all patients with an ICD 9 or ICD 10 code for RA who were tested with the commercial Vectra^®^DA test before June 2017 as part of routine care by US rheumatologists. Serum specimens had been submitted to a Clinical Laboratory Improvement Amendment (CLIA)-certified, New York State and College of American Pathologists (CAP)-accredited laboratory of Crescendo Bioscience for routine commercial testing. MBDA scores were de-identified for inclusion in the present research study. Patients >89 years old were excluded as age ≥90 years is considered personally identifying information. Only the first test result was included for patients who had been tested more than once.

REFERENCES

1. Curtis JR, van der Helm-van Mil AH, Knevel R, et al. Validation of a novel multibiomarker test to assess rheumatoid arthritis disease activity. Arthritis Care Res 2012; 64(12): 1794-803.
2. https://www.corrona.org/certain-rheumatoid-arthritis-registry
3. Brahe CH, Østergaard M, Johansen J, et al. Predictive value of a multi-biomarker disease activity (MBDA) score for clinical remission and radiographic progression in patients with early rheumatoid arthritis (RA) – a posthoc study of the OPERA trial. Scand J Rheumatol 2018 July 9: 1-8. doi: 10.1080/03009742.2018.1464206. [Epub ahead of print].
4. Iannaccone CK, Lee YC, Cui J et al. Using genetic and clinical data to understand response to disease-modifying anti-rheumatic drug therapy: data from the Brigham and Women's Hospital Rheumatoid Arthritis Sequential Study. Rheumatology 2011; 50: 40-6.

**Supplemental Table S1.** The clinical and demographic characteristics of the various healthy and RA study cohorts.

|  | **5 Studies/Registries (RA) (N=1,411)** | | **Commercial Tests (RA)**  **(N=325,781)** | | **Healthy Normal**  **(N=318)** | |
| --- | --- | --- | --- | --- | --- | --- |
| **Characteristics** | **N** | **Mean (SD) or**  **N (%)** | **N** | **Mean (SD) or**  **N (%)** | **N** | **Mean (SD) or**  **N (%)** |
| Age (years) | 1,411 | 57.3 (13.4) | 325,781 | 57.6 (14.1) | 318 | 53.1 (13.3) |
| Sex | | | | | | |
| Female | 1,411 | 1,098 (77.8%) | 325,781 | 256,470 (78.7%) | 318 | 203 (63.8%) |
| Male |  | 313 (22.2%) |  | 69,311 (21.3%) |  | 115 (36.2%) |
| Disease duration (yrs) | 1,125 | 12.8 (11.9) | 0 | NA | 0 | NA |
| RF/anti-CCP | | | | | | |
| Both negative | 1,402 | 346 (24.7%) | 0 | NA | 0 | NA |
| Both positive |  | 767 (54.7%) |  | NA |  | NA |
| Either positive |  | 289 (20.6%) |  | NA |  | NA |
| BMI (kg/m^2^) | 1,411 | 28.0 (6.1) | 0 | NA | 318 | 28.4 (6.6) |
| TJC 28 | 1,411 | 6.7 (7.3) | 0 | NA | 0 | NA |
| SJC 28 | 1,411 | 6.1 (6.3) | 0 | NA | 0 | NA |
| Patient Global (mm) | 1,389 | 19.8 (23.5) | 0 | NA | 0 | NA |
| ESR (mm/hr) | 1,411 | 22.6 (16.9) | 0 | NA | 0 | NA |
| CRP (mg/L) | 1,411 | 12.1 (22.6) | 0 | NA | 0 | NA |
| DAS28-ESR | 507 | 3.8 (1.5) | 0 | NA | 0 | NA |
| DAS28-CRP | 1,389 | 3.9 (1.6) | 0 | NA | 0 | NA |
| MBDA score | 1,389 | 43.2 (16.9) | 325,781 | 40.9 (15.3) | 318 | 31.0 (13.6) |
| Serum leptin (ng/mL) | 1,388 | 24.9 (23.8) | 325,781 | 31.8 (29.8) | 318 | 20.1 (21.5) |
| Total Sharp score | 579 | 23.6 (44.7) | 0 | NA | 0 | NA |

The 5 clinical trials/registries are BRASS, Corrona-CERTAIN, InFoRM, RACER, OPERA. InFoRM: Index For Rheumatoid Arthritis Measurement; RACER: Rheumatoid Arthritis Comparative Effectiveness Research; Corrona-CERTAIN: Comparative Effectiveness Registry to Study Therapies for Arthritis and Inflammatory CoNditions; OPERA: OPtimized Treatment Algorithm in Early Rheumatoid Arthritis; BRASS: Brigham Rheumatoid Arthritis Sequential Study; CRP: C-reactive protein, performed in a clinical laboratory; DAS28: disease activity score using 28 joints; MBDA: multi-biomarker disease activity; SJC: swollen joint count for 28 joints; TJC: tender joint count for 28 joints.

**Supplemental Table S2.** Baseline characteristics for the five cohorts comprising the clinical trial/registry cohort (total N=1411).

|  | **BRASS-047-CL-01 N=424** | | **CERTAIN-091-CL-01 N=105** | | **InFoRM**  **N=382** | | **OPERA-073-CL-01**  **N=168** | | **RACER-048-CL-01**  **N=332** | |
| --- | --- | --- | --- | --- | --- | --- | --- | --- | --- | --- |
| **Characteristic** | **N** | **Mean (SD) or**  **N (%)** | **N** | **Mean (SD) or**  **N (%)** | **N** | **Mean (SD) or**  **N (%)** | **N** | **Mean (SD) or**  **N (%)** | **N** | **Mean (SD) or**  **N (%)** |
| Age | 424 | 56.3 (12.7) | 105 | 53.6 (10.7) | 382 | 60.0 (13.2) | 168 | 52.8 (14.7) | 332 | 58.7 (13.8) |
| Sex | | | | | | | | | | |
| Female | 424 | 347 (81.8%) | 105 | 80 (76.2%) | 382 | 290 (75.9%) | 168 | 112 (66.7%) | 332 | 269 (81.0%) |
| Male |  | 77 (18.2%) |  | 25 (23.8%) |  | 92 (24.1%) |  | 56 (33.3%) |  | 63 (19.0%) |
| Disease duration (yrs) | 424 | 13.7 (11.8) | 105 | 4.5 (5.5) | 265 | 14.2 (12.1) | NA | NA | 331 | 13.1 (12.2) |
| RF/anti-CCP | | | | | | | | | | |
| Both negative | 419 | 102 (24.3%) | 105 | 22 (21.0%) | 261 | 75 (19.8%) | 168 | 38 (22.6%) | 332 | 109 (32.8%) |
| Both positive |  | 242 (57.8%) |  | 62 (59.0%) |  | 226 (59.8%) |  | 105 (62.5%) |  | 132 (39.8%) |
| Either positive |  | 75 (17.9%) |  | 21 (20.0%) |  | 77 (20.4%) |  | 25 (14.9%) |  | 91 (27.4%) |
| BMI (kg/m^2^) | 424 | 26.5 (5.1) | 105 | 29.2 (7.0) | 382 | 29.4 (7.1) | 168 | 25.5 (4.3) | 332 | 29.0 (5.9) |
| TJC 28 | 424 | 8.2 (7.8) | 105 | 9.7 (6.5) | 382 | 4.9 (6.6) | 168 | 11.6 (6.5) | 332 | 3.5 (5.6) |
| SJC 28 | 424 | 7.9 (7.0) | 105 | 7.4 (4.5) | 382 | 3.5 (4.7) | 168 | 9.6 (6.2) | 332 | 4.4 (5.7) |
| Patient global (mm) | 402 | 29.4 (24.0) | 105 | 4.9 (2.3) | 382 | 33.5 (27.5) | 168 | 6.3 (2.6) | 332 | 4.0 (2.6) |
| ESR (mm/hr) | NA | NA | NA | NA | 381 | 22.4 (16.2) | NA | NA | 126 | 23.2 (18.9) |
| CRP (mg/L) | 424 | 9.8 (21.5) | 105 | 11.3 (14.7) | 382 | 7.0 (10.4) | 168 | 32.6 (37.9) | 332 | 10.9 (20.5) |
| DAS28 | NA | NA | NA | NA | 381 | 3.8 (1.5) | NA | NA | 126 | 3.8 (1.7) |
| DAS28-CRP | 402 | 3.9 (1.5) | 105 | 4.7 (1.0) | 382 | 3.3 (1.4) | 168 | 5.6 (1.1) | 332 | 3.4 (1.4) |
| MBDA score | 424 | 40.0 (16.5) | 32 | 47.3 (18.7) | 382 | 40.6 (13.6) | 168 | 58.5 (15.7) | 332 | 42.3 (17.0) |
| Serum leptin (pg/mL) | 424 | 20227.0 (20651.0) | 32 | 25275.9 (22760.3) | 382 | 31196.4 (27535.7) | 168 | 16285.3 (17365.5) | 332 | 28000.6 (23614.4) |
| Total Sharp score | 419 | 31.0 (50.5) | NA | NA | NA | NA | 160 | 4.3 (5.7) | NA | NA |

CRP: C-reactive protein, performed in a clinical laboratory; DAS28: disease activity score using 28 joints; MBDA: multi-biomarker disease activity; SJC, swollen joint count for 28 joints; TJC, tender joint count for 28 joints.

**Supplemental Table S3.** Cross-classification of 325,781 RA patients into high, moderate and low MBDA categories using original MBDA score and leptin-adjusted MBDA score.

| **Original MBDA Score** | **Leptin-adjusted MBDA Score** | | |
| --- | --- | --- | --- |
|  | Low | **Moderate** | **High** |
| Low | 55686 (76%) | 17163 (24%) | 182 (0.2%) |
| Moderate | 11339 (9%) | 97495 (79%) | 14206 (12%) |
| High | 0 (0%) | 27862 (21%) | 101848 (79%) |

Categories for low (<30), moderate (30−44), high (>44) disease activity apply to the original and leptin-adjusted MBDA scores. MBDA: multi-biomarker disease activity.

**Supplemental Figure S1.** Relationship between MBDA score and serum leptin levels in 325,781 RA patients categorized by age and leptin level.

_
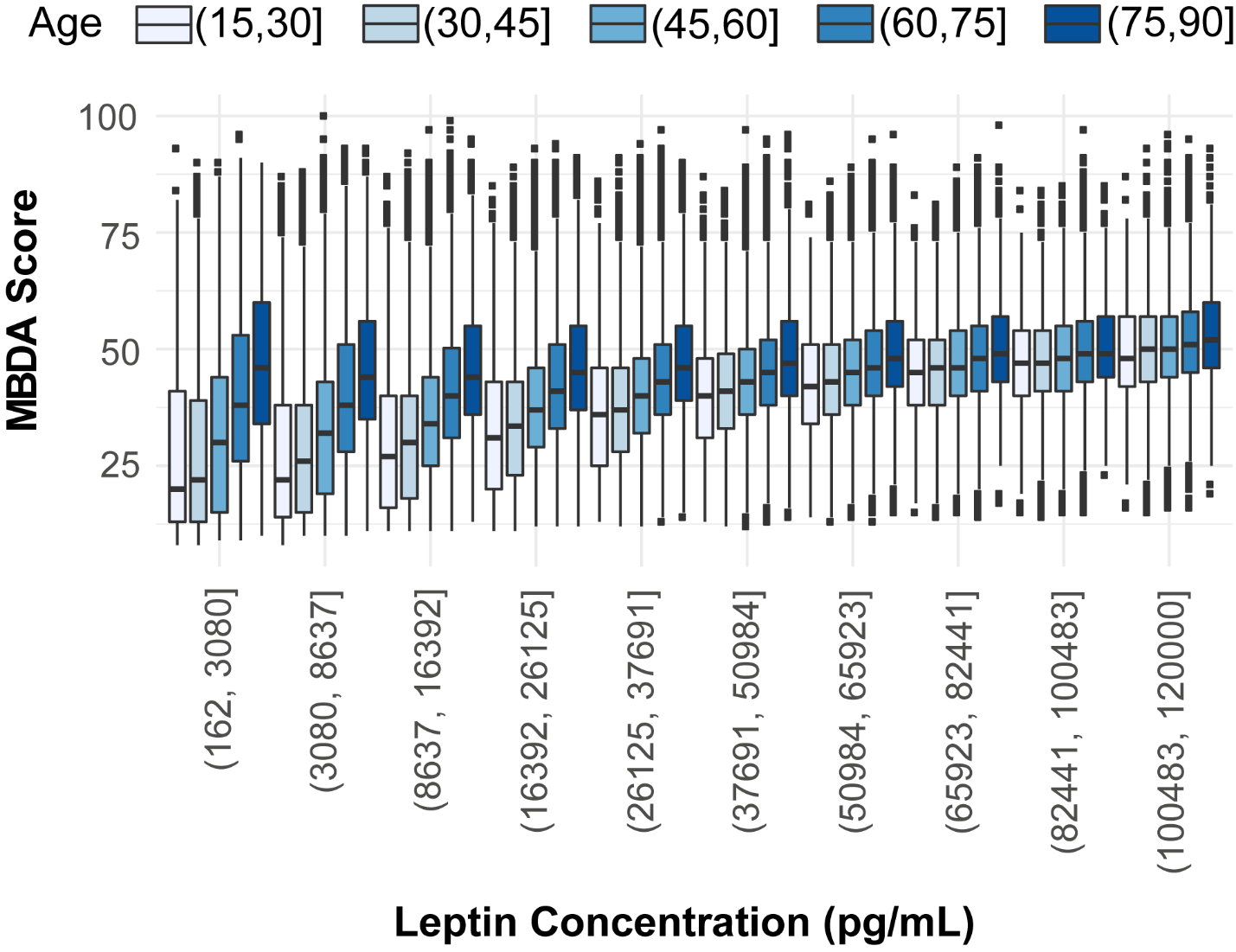
_

**Supplemental Figure S2.** Distribution of differences between original MDBA score and leptin-adjusted MBDA scores.


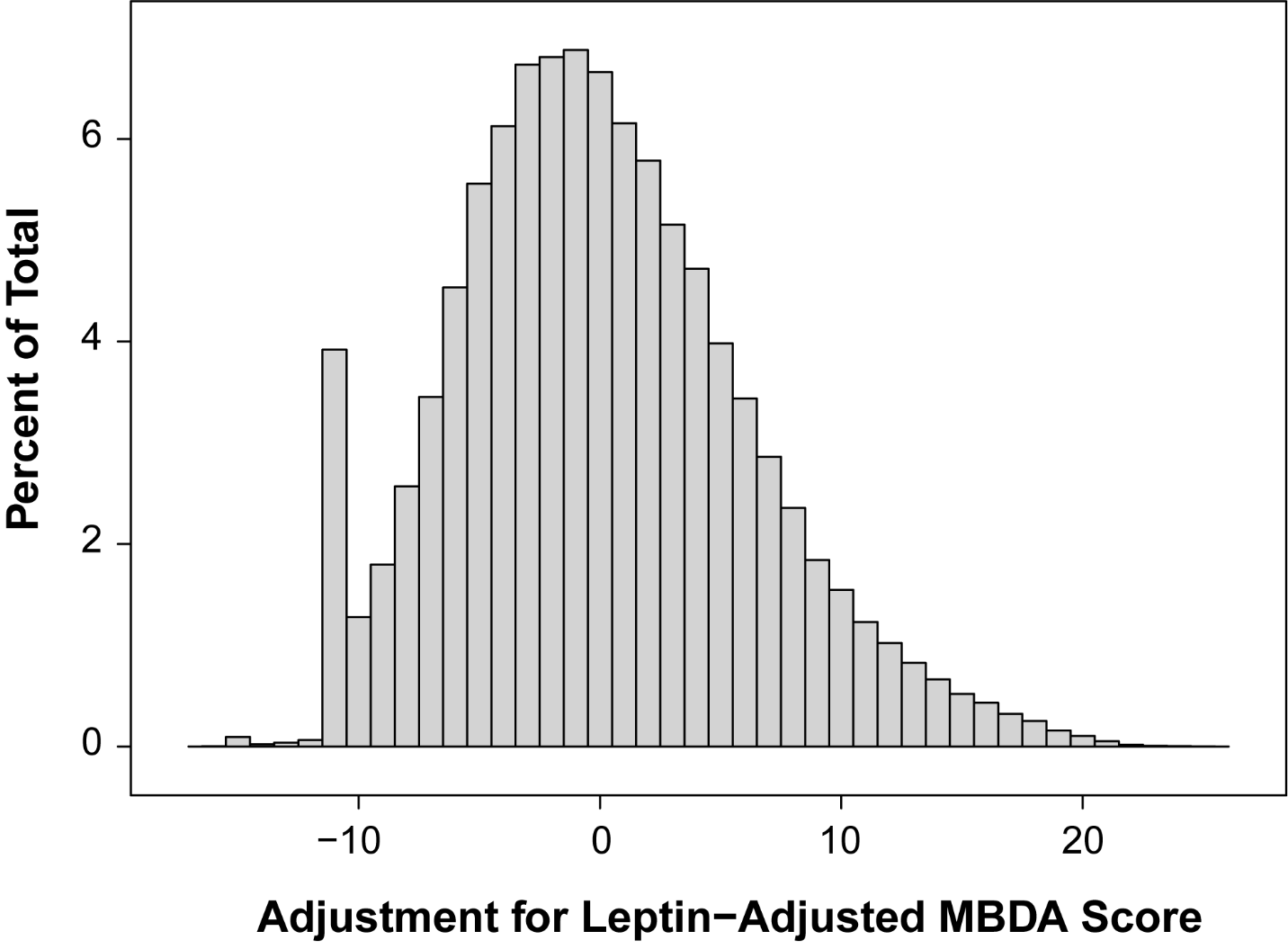


The upper limit of quantitation for leptin concentration in the MBDA test is 120 ng/ml.
